# Editorial: Virtual Environments as Study Platforms for Realistic Human Behavior

**DOI:** 10.3389/fpsyg.2016.01361

**Published:** 2016-09-27

**Authors:** Joshua C. Poore, Clint Bowers

**Affiliations:** ^1^Charles Stark Draper Laboratory, Information and Decision Sciences, Human Centered EngineeringCambridge, MA, USA; ^2^Department of Psychology, University of Central FloridaOrlando, FL, USA

**Keywords:** virtual environments, human behavior, virtual, test stimuli, realistic human behavior

## Introduction to the research topic

As of 2013, US persons spend an average of 37 h per month using smartphone applications, another 27 h using the internet on personal computers, and 133 h watching live television (Nielsen, 2014). Coupled with the estimated 64% of US persons that own a smartphone (Pew, 2015), people are spending a large sum of their waking lives plugged into virtualized, but very real, software-mediated environments. In these cases, and where virtual environments (VEs) are meant to emulate the real-world for gaming and simulation, the psychological sciences now have an unprecedented opportunity to use them as mediums for understanding humans in the real-world (Bowers et al., 2008). VEs can be used to capture high fidelity data about human behavior in a way that far exceeds what can be captured in existing laboratory and daily experience research methods. The current challenge is to harness this data for observing and modeling how people use to manipulate their environment, forage for information, and work with people in organic, unstructured interactions. This research topic introduces new approaches and use-cases for how VEs can enhance the ecological validity of laboratory studies, and the generalizable study of human behavior in naturalistic, real-world environments, even though they may be virtual.

## State of the practice and the cutting-edge

A key consideration in using VEs as real-world analogs is whether they are, in fact, valid analogs. Bomberi et al. provide a review on VEs used for research and training. Where VEs have historically lacked sufficient resolution, fidelity, and interactivity, modern gains in computing and it application into gaming, simulation, and virtual reality capabilities are progressing in leaps. Modern VE platforms are far more successful than previous iterations for inducing meaningful experiences and eliciting sincere responses from participants. Topic articles also compare virtual reality platforms to more traditional laboratory stimulus presentation modes, namely video media, finding that VEs offer richer experiences for subjects (Dibbets and Schulte-Ostermann). van der Ham et al. also provides a comparison between real- and virtual-environments for use in spatial navigation training. This work provides a compelling case of the utility of VEs in the ability to capture valuable process-level data not previously available.

Other studies presented in this topic demonstrate how VEs can be useful for extending the translational value of research, exploring key domains like post-traumatic stress syndrome (Dibbets and Schulte-Ostermann; Highland et al.) and social anxiety (Shiban et al). Shiban et al. also make an interesting case for utilizing VEs for translational research in these phenomena to bridge the gap between human and animal research, in generative ways never before possible.

This topic also explores new methods for extracting data from VEs for new inquiry. Emerging trends in open-source software, open-architecture, and application program interfaces (APIs) hold promise for access to rich data about behavioral process from VEs for use in cognitive and behavioral modeling (Poore et al., 2016). Stone et al. for example, demonstrate that simulation instrumentation can be used for harvesting useful data for experimental research and algorithms. Using an instrumented driving simulator, they identify specific driving conditions that are most likely to be challenging for drivers under the influence of benzodiazepines.

Other topic authors explore generalized modeling approaches that can be applied to newly available data extracted from VEs. Cipresso offers a multi-tier modeling approach for behavioral dynamics that is specifically suited for capitalizing on data from virtual environments, and feedback from these environments. Buckmann et al. make use of simple virtual environments with haptic interfaces and identify methods for quantifying kinematic exploratory behavior with novel objects. By using data collected through haptic interfaces they not only demonstrate methods for quantifying efficiency in learning, but modeling what was learned and how much was learned through exploration. Mariano et al. explore novel implementations of non-parametric systems-level approaches to capture the strategies with which people interact with software tools to complete tasks, even with very little training. These models offer a content agnostic framework for understanding *how* people perform tasks within software environments, which relates to both task-related cognitive states and personality characteristics.

## Future directions and research agendas

Understanding human behavior in real-world, dynamic environments is challenging. Difficulties in collecting traditional measures (e.g., survey, psychophysiologic measures) at a meaningful sampling rate, and collecting enough data to be able to control for extraneous stimuli have been major impediments to generalizing research findings outside the laboratory. As VEs become more ubiquitous in their use, new opportunities for emulating the real-world with meaningful fidelity in the lab are emerging, as are quantifying the sum total of data within these environments from entities, ecology, to their physics. Where VEs are not meant to emulate the real-world, but include virtualized assets that people use in their daily life (e.g., analytic, professional, and social media software) we will be able to measure and model how people interact through email, do work through a suite of applications, and even operate their vehicles. Just as VEs offer realistic analogs of the world we live in now, the way we interact with them and data we can collect from them are analogous to the world we will come to know in the very near term. The methods and approaches collected in this topic will be useful as the psychological sciences continue to blend their skill sets with those of the data and computer sciences. Pushing innovation in how VEs can be exploited for research purposes will prepare us to fully on day capture the shared virtualized environments in which people live and do their work. Through these innovations, a new understanding of even the most basic cognitive and affective mechanisms will be 1 day fully obtainable and interpretable in their native context.

## Author contributions

All authors listed, have made substantial, direct and intellectual contribution to the work, and approved it for publication.

### Conflict of interest statement

The authors declare that the research was conducted in the absence of any commercial or financial relationships that could be construed as a potential conflict of interest.
